# Rotation of Magnetization Derived from Brownian Relaxation in Magnetic Fluids of Different Viscosity Evaluated by Dynamic Hysteresis Measurements over a Wide Frequency Range

**DOI:** 10.3390/nano6090170

**Published:** 2016-09-10

**Authors:** Satoshi Ota, Ryoichi Kitaguchi, Ryoji Takeda, Tsutomu Yamada, Yasushi Takemura

**Affiliations:** 1 Department of Electrical and Electronic Engineering, Shizuoka University, Hamamatsu 432-8561, Japan; 2 Department of Electrical and Computer Engineering, Yokohama National University, Yokohama 240-8501, Japan; kitaguchi-ryoichi-kj@ynu.jp (R.K.); takeda-ryoji-mh@ynu.jp (R.T.); yamada@ynu.ac.jp (T.Y.); takemura@ynu.ac.jp (Y.T.)

**Keywords:** magnetic nanoparticles, magnetic relaxation, hysteresis loop, magnetic fluid, viscosity

## Abstract

The dependence of magnetic relaxation on particle parameters, such as the size and anisotropy, has been conventionally discussed. In addition, the influences of external conditions, such as the intensity and frequency of the applied field, the surrounding viscosity, and the temperature on the magnetic relaxation have been researched. According to one of the basic theories regarding magnetic relaxation, the faster type of relaxation dominates the process. However, in this study, we reveal that Brownian and Néel relaxations coexist and that Brownian relaxation can occur after Néel relaxation despite having a longer relaxation time. To understand the mechanisms of Brownian rotation, alternating current (AC) hysteresis loops were measured in magnetic fluids of different viscosities. These loops conveyed the amplitude and phase delay of the magnetization. In addition, the intrinsic loss power (ILP) was calculated using the area of the AC hysteresis loops. The ILP also showed the magnetization response regarding the magnetic relaxation over a wide frequency range. To develop biomedical applications of magnetic nanoparticles, such as hyperthermia and magnetic particle imaging, it is necessary to understand the mechanisms of magnetic relaxation.

## 1. Introduction

Magnetic nanoparticles (MNPs) attract attention for biomedical applications such as hyperthermia treatment and magnetic particle imaging (MPI), exhibiting potential for therapeutic and diagnostic applications [1,2]. During their synthesis and their introduction into the human body and in an in vivo environment such as blood, MNPs are dispersed in liquid. In addition to Néel relaxation, it is important to understand the effect of Brownian relaxation. The Brownian and Néel relaxation times are given as:

(1) $$\tau_{B}=\frac{3\eta V_{H}}{k_{B}T}$$

(2) $$\tau_{N}=\tau_{0}\exp\left(\frac{KV_{M}}{k_{B}T}\right)$$

respectively, where η is the viscosity, *V*_H_ is the hydrodynamic volume of MNPs, τ_0_ is the attempt time of ~10^−9^ s associated with gyromagnetic procession, *K* is the magnetic anisotropic constant, *V*_M_ is the volume of the core particle (monodomain), *k*_B_ is the Boltzmann constant 1.38 × 10^−23^ J/K, and *T* is the temperature in Kelvin [3,4]. In the conventional theory, when Brownian and Néel relaxations occur in parallel, the relaxation time τ is given as the effective one expressed as [3]:

(3) $$\frac{1}{\tau }=\frac{1}{\tau_{B}}+\frac{1}{\tau_{N}}$$

In addition, when the applied field frequency *f* is the most suitable for the relaxation time τ, *f* and τ are related as follows [3]:

(4) 2πfτ=1

To understand the mechanisms of magnetic relaxation, the susceptibility has been measured [5,6,7]. The susceptibility measurements also reflect the MNP size distribution and the particle rotation associated with the MNP structures [6,7]. In addition, a reduction of the Brownian and Néel relaxation times with an increasing applied-field intensity was measured according to the alternating current (AC) susceptibility [8]. The equation for the influence of the Brownian relaxation time on the applied field is estimated as [9]:

(5) $$\frac{1}{\tau_{B,\mathrm{eff}}}=\frac{1}{\tau_{B}}\sqrt{1+0.21\xi }$$

where τ_B,eff_ is the Brownian relaxation time considering the field intensity, and ξ = *M*_s_*H*/*k*_B_*T* is the ratio of the external field energy to the thermal energy. *M*_s_ is the saturation magnetization, and *H* is the field intensity. Equation (5) shows that the Brownian relaxation time decreases as the field intensity increases. In this study, AC hysteresis loops were measured to reveal the mechanisms of the particle and magnetization rotations associated with magnetic relaxations in magnetic fluids having different viscosities. The susceptibility characterizes the magnetic properties only when the magnetization has the maximum value as a response to the maximum intensity of the applied field. On the other hand, the AC hysteresis loops indicate that the magnetization-reversal process changes with the field intensity, which is a feature not exhibited by the susceptibility. The time-evolution response of magnetization is an important feature that indicates the mechanisms of magnetic relaxation. In previous studies, AC hysteresis loops have been measured at frequencies of 394 and 785 kHz or in the frequency range extending down to 1 kHz [10,11]. In addition, the influences of the MNP concentration and viscosity on heat dissipation have been evaluated using AC hysteresis loops [11,12]. Temperature-rise measurements have shown that high-anisotropy MNPs are significantly influenced by the fluid viscosity, as the effect of Brownian relaxation dominates the heating power, whereas the influence of the viscosity on low-anisotropy MNPs is negligible because the Néel relaxation dominates the heating power [13]. Highly accurate measurement systems—including pick-up coils, which have adjustable parameters such as the number of turns, shape, and size—enable AC hysteresis measurements over a wide frequency range down to 10 Hz. This is essential for clarifying the dynamic magnetization of magnetic fluids. A signal of only a few dozen microvolts can thus be detected. The AC hysteresis loop of a liquid sample was measured by this system, in addition to that of a solid sample [14]. In the present study, the dependence of the heat dissipation on the viscosity was measured in a low frequency range (10 Hz–300 kHz) compared with the measurement frequency of 700 kHz used in a conventional study [15]. Measurement over a wide frequency range for different viscosities is necessary to understand the mechanisms of magnetic relaxation. To evaluate the frequency dependence of magnetic relaxation, the intrinsic loss power (ILP) was estimated according to the area of the AC hysteresis loops. The ILP is given as

(6) $$\mathrm{ILP}=\frac{\mu_{0}\pi }{\rho }\chi \prime \prime$$

where μ_0_ is the permeability of free space, χ″ is the imaginary component of susceptibility, and ρ is the MNP mass density [3,11,16]. Equation (6) shows that the ILP is significantly similar to χ″ as a criteria for measuring the mechanism of magnetic relaxation. The relaxation mechanism in a higher field than the field intensity applicable to the linear response theory (LRT) is evaluated by the ILP estimated from AC hysteresis loops. Conversely, especially in the evaluation of the phase delay associated with magnetic relaxation, the susceptibility is not suitable for measurement in a higher field than the field intensity applicable to the LRT. The relationships of the susceptibility with the phase delay and magnetization are defined on the basis of the LRT [17]. The measured MNP for the applied-field intensity used in this study is not applicable to the LRT. In the measurement of AC hysteresis loops, the waveform of the magnetization signal was not sinusoidal, despite the sinusoidal applied field. In addition, the shapes of the AC hysteresis loops were not fully ellipsoidal, whereas those of the AC hysteresis loops calculated on the basis of the LRT are fully ellipsoidal [17]. The linearity of direct current (DC) magnetization response is lower than that of AC magnetization response because a cycle of the applied field in the DC hysteresis measurement is much longer than that in AC hysteresis measurement. In the DC hysteresis measurement, the field is applied for the period sufficiently longer than the relaxation time. The period is also longer than that in the AC hysteresis measurement in this study. In addition to AC hysteresis loops, DC hysteresis loops indicate the magnetization response. It has been shown that the particle rotates and aligns with the direction of the external field in a medium because of the measurement of DC hysteresis loops [18]. Moreover, according to the Stoner-Wohlfarth model, the degree of magnetization rotation depends on the Zeeman and anisotropic energies, which are given as:

(7) $$E_{h}=\mu_{0}MV_{M}H\cos\left(\varphi -\theta \right)$$

(8) $$E_{a}=KV_{M}\sin^{2}\left(\theta \right)$$

where *M* is the volume magnetization, φ is the angle between the easy axis and the magnetic field, and θ is the angle between the easy axis and the magnetization [19]. The AC hysteresis measurement allows the determination of the magnetic properties of the MNPs suspended in a liquid, fixed to inhibit particle rotation, and added in a cellular environment [20,21,22].

## 2. Materials and Methods

A water-based Fe_3_O_4_ nanoparticle fluid, which is commercially distributed as M-300 by Sigma Hi-chemical Inc. (Chigasaki, Kanagawa, Japan), was used. The particles were coated with α-olefin sulphonic acid sodium. Their core diameter was 11 ± 3 nm, as determined by transmission electron microscopy. Their hydrodynamic diameter was 52 ± 12 nm, as measured by dynamic light scattering. The concentration of this sample was adjusted to 90 mg-Fe/mL. The MNPs were aggregated in the fluid as hydrodynamic particles. The number of hydrodynamic particles was ~32 × 10^19^ per unit volume. The surface-to-surface distance between the hydrodynamic particles was 94 nm, as estimated according to the hydrodynamic diameter of 52 nm and the concentration of 90 mg-Fe/mL. The distance between the hydrodynamic particles is equal to the inter-aggregate distance shown by Ovejero et al. [23]. The cubically arrayed hydrodynamic particles were conditioned for this calculation, and the smallest distance between the particles was calculated as the inter-aggregate distance. The fluid viscosity was adjusted to 2.4, 4.7, 12, or 93 mPa·s by changing the weight ratio of MNPs, deionized water, and glycerol. The viscosity of the fluid was measured using a viscosity meter (microVISC, Viscotech Co., Ltd., Tokyo, Japan). The sample viscosities were the values at the temperature of 25 °C because the DC and AC hysteresis loops were measured at 25 °C.

In the circuit used for measuring the AC hysteresis loops, the electromagnetic induction at a resonance point was measured by connecting a capacitor suited to the frequency of the generated AC [18]. The coils used for detection consisted of pick-up and cancel coils located inside of an excitation coil. The AC hysteresis loops were measured in the frequency range of 10 Hz–300 kHz at a field intensity of 4 kA/m. The dependence of the AC hysteresis properties on the applied field intensity have also been measured [20]. DC hysteresis loops were measured using a vibrating sample magnetometer (VSM) at 25 °C. The samples used in the measurements of the DC and AC hysteresis loops had a volume of 0.2 mL and were encapsulated in a cylindrical tube with a diameter of 10 mm. The magnetization was quantified using its value, normalized by the saturation magnetization, with the unit of *M*/*M*_s_, because the relative value directly shows the degree of magnetization rotation. For the normalization, the saturation magnetizations of the measured samples were estimated by fitting the measured DC magnetization curve to the magnetization curve calculated using the Langevin function. The saturation magnetization per unit weight of the measured MNP was 72 Am^2^/kg-Fe. The ILP was calculated using this value of the saturation magnetization per unit weight.

## 3. Results and Discussion

**Figure 1:**
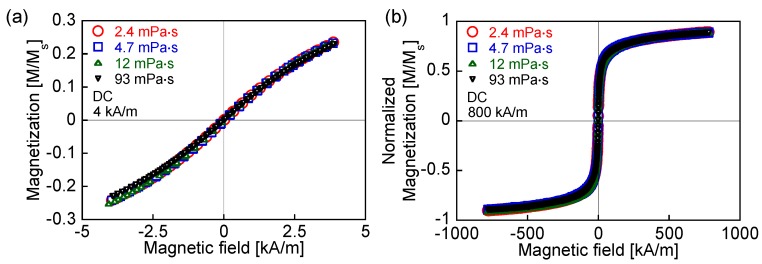
Direct current (DC) hysteresis loops covering field intensities of (**a**) 0–4 kA/m and (**b**) 0–800 kA/m for viscosities of 2.4, 4.7, 12, and 93 mPa·s.

Figure 1 shows the dependence of the DC hysteresis loops on the viscosity at maximum field intensities of 4 and 800 kA/m. The difference in the magnetization for the different viscosities is marginal. The rotational degree of magnetization, which is derived from the particle rotation, is constant with changes in the viscosity when a DC field is applied, because the frictional energy associated with the viscosity is time-dependent:

(9) $$E_{f}\left(t\right)=6\eta V_{H}\frac{d\theta \left(t\right)}{dt}$$

where *d*θ(*t*)/*dt* is the angular velocity of the particle [24]. The DC field is applied over a period that exceeds the relaxation time of the measured MNP. In addition, the value of the saturation magnetization does not change according to the viscosity because of Equation (9).

**Figure 2:**
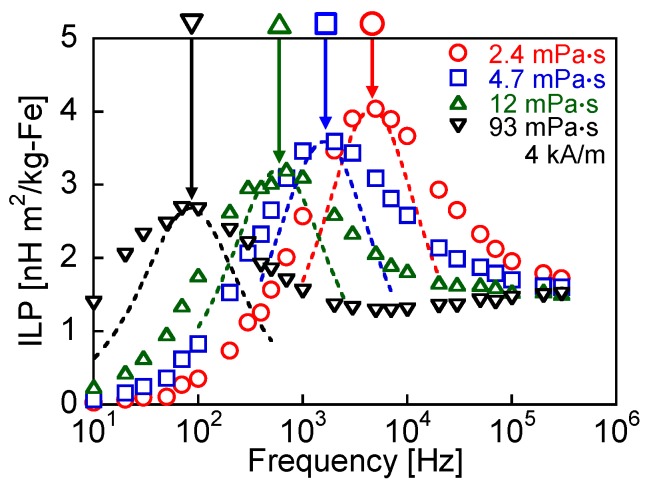
Frequency dependence of the intrinsic loss power (ILP), estimated from the area of alternating current (AC) hysteresis loops for viscosities of 2.4, 4.7, 12, and 93 mPa·s. The corresponding measured ILP peaks are 4.6, 1.6, 0.6, and 0.084 kHz, respectively. The dotted curves are the fitting curves calculated using Equation (10) and the normalization of the calculated ILPs with respect to the measured ILPs at the relaxation peak frequencies.

Figure 2 shows the frequency dependence of the ILP for each viscosity. The peaks in the ILP indicate the peaks of Brownian relaxation. It has been confirmed that the low-frequency ILP peak was not observed in MNPs fixed with an epoxy bond, in which only Néel relaxation occurs [11]. Table 1 shows the peak frequencies and relaxation times of Brownian and Néel relaxation. The theoretical values were calculated using Equations (1), (2) and (4). With respect to Brownian relaxation, the measured values were estimated according to Figure 2. When the relaxation time is defined as the effective relaxation time shown in Equation (3), the single magnetic relaxation of the shorter relaxation time dominates. The peak frequency of ILP shows the relaxation time (Equation (4)). A single peak is observed when the single magnetic relaxation dominates, and the magnetic relaxation of the shorter relaxation time is indicated as the ILP peak. However, in Figure 2, the ILP peak derived from the Brownian relaxation is observed, in spite of the longer Brownian relaxation time compared with the Néel relaxation time. It is shown that Brownian relaxation coexists with Néel relaxation. The coexistence of the relaxations is discussed according to the evaluation of AC hysteresis loops. The theoretical peak frequency of Néel relaxation is significantly higher than the measurement frequency range (Table 1). Except for the viscosity of 93 mPa·s, the ILP decreased with the increasing frequency at measurement frequencies higher than the Brownian relaxation peak because the decrease of the ILP derived from Brownian relaxation was more significant than its increase derived from Néel relaxation. In the frequency range higher than 10 kHz, the ILP for the viscosity of 93 mPa·s monotonically increased with the frequency because of the phase delay associated with Néel relaxation. With respect to Brownian relaxation, the theoretical frequencies are lower than the measured frequencies because an external field enhances particle rotation and shortens the Brownian relaxation time [9]. It has also been shown that both the Brownian and Néel relaxation times depend on the field intensity and, in particular, the dependence of the Néel relaxation time on the field intensity is significant [25]. The relaxation time under an applied field is shorter than the zero-field relaxation time. The frequency of the measured ILP peak decreases with increasing viscosity, which agrees with the conventional theory because the Brownian relaxation time increases with increasing viscosity (Equation (1)). However, the ILPs at the relaxation peak frequencies decreased with increasing viscosity. In the conventional theory, the ILP at the relaxation peak frequency is constant with changes in the viscosity because, according to Equation (6), the ILP is proportional to the imaginary component χ″ of the susceptibility, which is given as:

(10) $$\chi \prime \prime =\chi_{0}\frac{2\pi f\tau }{1+{\left(2\pi f\tau \right)}^{2}}$$

where χ_0_ is the equilibrium susceptibility [17]. χ″ is constant at the relaxation peak frequency (Equations (4) and (10)). The susceptibility and phase delay of the magnetization from the applied field are respectively given as [17]:

(11) $$\chi =\frac{\chi_{0}}{\sqrt{1+{\left(2\pi f\tau \right)}^{2}}}$$

(12) $$\sigma =\sin^{-1}\left(\frac{2\pi f\tau }{\sqrt{1+{\left(2\pi f\tau \right)}^{2}}}\right)$$

The dotted curves in Figure 2 are the fitting curves calculated using Equation (10) and the normalization of the calculated ILPs with respect to the measured ILPs at the relaxation peak frequencies because the ILP is proportional to χ″ (Equation (6)). The shapes of these dotted curves are sharper than those of the measured ILP because the particle size distribution is not considered in the theoretical estimation using Equation (10). The real particles are composed of particles with broadly distributed sizes that show different relaxation times. The difference between the experimental plots and the fitting curves increases, particularly at high frequencies, because the effects of both Brownian and Néel relaxations are associated with the experimental ILP, whereas only the effect of Brownian relaxation is considered in the fitting curves. In addition, a dipolar interaction occurs for the concentration of 90 mg-Fe/mL and increases the relaxation time [11]. However, the dependence of the magnetic relaxation on the fluid viscosity can be measured without considering the effects of dipolar interaction because the effects of dipolar interaction are constant at the same concentration. In the previous study, the reduction of the Brownian relaxation time with decreasing particle concentration was observed [11]. The Brownian relaxation time for the lowest concentration of 37 mg-Fe/mL was 16 μs, which is significantly longer than the Néel relaxation time of 6.5 ns. Thus, the Brownian relaxation time for the concentration of 90 mg-Fe/mL was also significantly longer than Néel relaxation time. In this study, the condition that the Néel relaxation time is shorter than the Brownian relaxation time is important. It is indicated that this condition is not changed by the effects of dipolar interaction because the Brownian relaxation time is significantly longer than Néel relaxation time.

**Figure 3:**
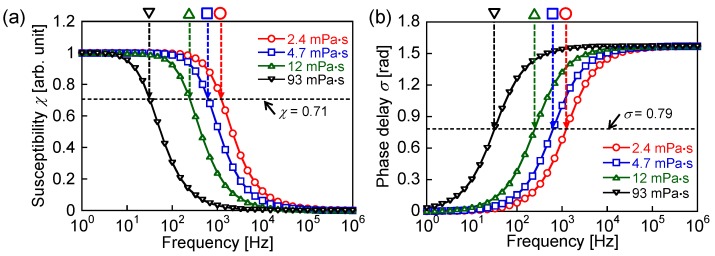
Theoretical (**a**) susceptibility and (**b**) phase delay of the magnetization, calculated by Equations (11) and (12), respectively, for viscosities of 2.4, 4.7, 12, and 93 mPa·s. The dotted lines show the susceptibility and phase delay for the conditions indicated by Equation (4). When these conditions are satisfied at the frequencies of the relaxation peaks, χ and σ are 0.71 and 0.79, respectively. The intersections of the dotted lines and the curves indicate the theoretical frequencies of the Brownian relaxation peaks for each viscosity, which are listed in Table 1.

**Figure 4:**
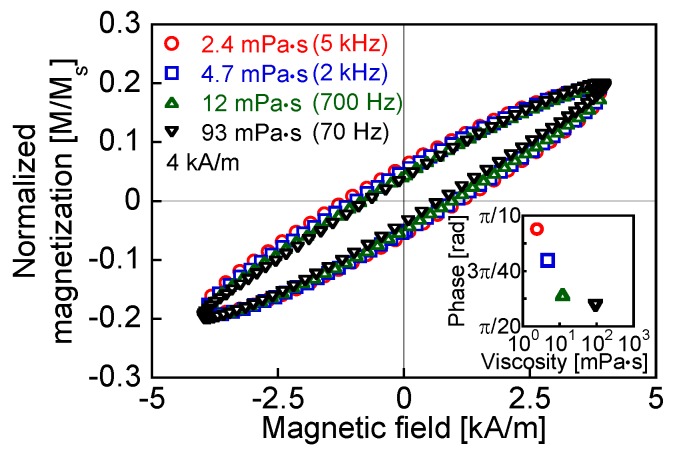
Measured AC hysteresis loops for viscosities of 2.4, 4.7, 12, and 93 mPa·s at frequencies close to the relaxation peaks shown in Table 1. The insets show the viscosity dependence of the phase delay derived from the coercivities of the AC hysteresis loops.

Figure 3 shows the theoretical susceptibility and phase delay of the magnetization, which were calculated using Equations (11) and (12), respectively. The intersection points of the dotted lines with the curves indicate the theoretical frequencies of the Brownian relaxation peaks for each viscosity, as listed in Table 1. The dotted lines in Figure 3 show the susceptibility (χ = 0.71) and phase delay (σ = 0.79) for the conditions indicated by Equation (4). The conditions indicated by Equation (4) are satisfied at the frequencies of the relaxation peaks. The magnetization is proportional to the susceptibility. Thus, in theory, according to Figure 3, the magnetization and phase delay are constants at the frequency of the relaxation peak. Figure 4 indicates the AC hysteresis loops at the frequencies close to the Brownian relaxation peaks shown in Table 1 for each viscosity. The rotational degree of magnetization is constant with changes in the viscosity, which agrees with Figure 3a. In contrast, the phase delay, which is indicated by the coercivity of the AC hysteresis loop, decreases with increasing viscosity in Figure 4. This disagrees with Figure 3b. The decrease in the ILPs at the relaxation peak frequencies in Figure 2 was due to the decrease of the phase delay shown in Figure 4.

**Figure 5:**
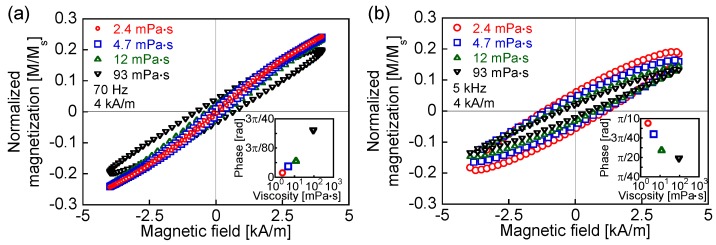
Measured AC hysteresis loops for viscosities of 2.4, 4.7, 12, and 93 mPa·s at frequencies close to the ILP peak for viscosities of (**a**) 93 and (**b**) 2.4 mPa·s at (**a**) 70 Hz and (**b**) 5 kHz, respectively. The insets show the dependence of the phase delay on the viscosity, which was derived from the coercivities of the AC hysteresis loops.

In theory, the susceptibility decreases and the phase delay increases as either the frequency or the viscosity increases. Figure 5a,b show AC hysteresis loops at the frequencies close to the measured ILP peaks for viscosities of 93 and 2.4 mPa·s as 70 Hz and 5 kHz, respectively. The reduction of the maximum magnetization is only marginal for viscosities of 2.4, 4.7, and 12 mPa·s, whereas for a viscosity of 93 mPa·s, the maximum magnetization is significantly lower than that for the other viscosities shown in Figure 5a. This is because the Brownian relaxation times for the lower viscosities are shorter than that for 93 mPa·s. The magnetization decreases with increasing viscosity in Figure 5b because the field was applied for periods shorter than the Brownian relaxation time. Moreover, as shown in Figure 5a, the phase delay increased with the viscosity because of the lower degree of particle rotation in the high-viscosity condition. This agrees with the predictions of the conventional theory shown in Figure 3b. In contrast, the phase delay decreases with increasing viscosity in Figure 5b, which is the opposite response of the magnetization to that expected from the conventional theory, as shown in Figure 3b. The conventional theory, represented by Equation (3), predicts that the faster relaxation should dominate [3,26]. On the other hand, the rotation of the magnetization in the measured fluid has contributions from both the magnetization and the particle rotations, which produces the opposite response to the one indicated above. The coexistence of Brownian and Néel relaxations has been demonstrated by comparing the MNPs in a liquid at 300 K with those in a frozen liquid at 250 K [27]. Furthermore, by the numerical simulation of the heat-dissipation efficiency, it has been shown that not only Néel relaxation but also Brownian relaxation occurs in a rotatable particle in a fluid [28]. The measurement of heat dissipation has also shown that there is a subsidiary contribution of Brownian relaxation in the heat dissipation that is mainly due to Néel relaxation [15].

First, at a frequency higher than the Brownian relaxation peak for the lowest viscosity, the contribution of the particle rotation to the magnetization is reduced with increasing viscosity because of the increasing relaxation time. The effect of the phase delay derived from particle rotation decreases with increasing viscosity. In addition, the phase delay derived from Néel relaxation is viscosity-independent. Thus, the combined phase delay, which is influenced by both the magnetization and the particle rotations, decreases with increasing viscosity at frequencies higher than the Brownian relaxation peak. The influence of the phase delay derived from the Brownian relaxation decreases at a high viscosity because of the lower degree of particle rotation. Moreover, the theoretical Néel relaxation peak frequency calculated using Equation (2) is 25 MHz (*K* = 11 kJ/m^3^), which is significantly higher than the Brownian relaxation peak frequency. This indicates that Brownian relaxation occurs after Néel relaxation, which has a shorter relaxation time than Brownian relaxation. Thus, at the peak frequency of Brownian relaxation, the phase delay decreases with increasing viscosity, in contrast to the conventional theory shown in Figure 3b (Figure 4). This is because the phase delay of magnetization increases with the increase of the frequency, whereas the phase delay of the particle rotation is constant at the frequency of the Brownian relaxation peak, which agrees with the conventional theory shown in Figure 3b. This also shows the coexistence of Brownian and Néel relaxations.

The magnetic responses of the MNPs from the relatively low field intensity used in this study are divided into the viscous modes suggested by Usov and Liubimov because the AC hysteresis loop was significantly influenced by the field frequency and fluid viscosity, which was indicated by the ILP derived from the area of the AC hysteresis loop (Figure 2) [29]. The dependence of the magnetization response on the viscosity is applicable regardless of the field intensity because the Brownian relaxation time shown in Equation (5) increases with the viscosity at a constant field intensity [9]. However, the magnetization responses at field intensities higher than the anisotropic field intensity, which is so high that the magnetization is close to saturation, differ from those at such low field intensities that the magnetization response shows the viscous mode [29]. Thus, the magnetization and particle rotations derived from magnetic relaxations at a field intensity lower than that of the anisotropic field can be modeled by the aforementioned evaluation of the phase delay combined with the rotational degree of magnetization depending on the viscosity.

**Figure 6:**
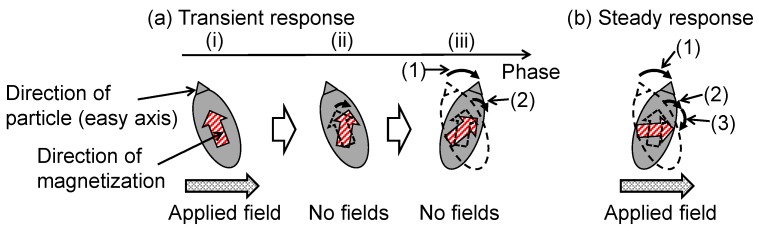
Schematic of the magnetic nanoparticle (MNP) magnetization and particle rotations in the (**a**) transient and (**b**) steady-state responses, under conditions where the Néel relaxation is faster than the Brownian relaxation. In the transient response, (**a-i**) a magnetic field is applied and the Zeeman energy is increased; (**a-ii**) The magnetization rotates owing to the increase of the Zeeman energy in phase (**a-i**); (**a-iii-1**) The particle rotates owing to the magnetic torque caused by the magnetization rotation in phase (**a-ii**); and (**a-iii-2**) the magnetization rotates owing to the decrease of the anisotropic energy caused by particle rotation; In the steady-state response, (**b-1**) particle rotation due to magnetic torque, (**b-2**) magnetization rotation due to the decrease of the anisotropic energy; and (**b-3**) magnetization rotation caused by the increase of the Zeeman energy occur.

Figure 6a,b shows the transient and steady-state responses, respectively, of the magnetization and particle rotations of an MNP under conditions where the Néel relaxation time is shorter than the Brownian relaxation time. These models are estimated according to the magnetization responses depending on the fluid viscosity used in this study and that in the solid state used in a previous study [11]. The models of magnetization and particle rotations shown in Figure 6 are evidenced by the magnetization responses measured by the AC hysteresis loops. The magnetization responses shown in the experiment of this study occur in the steady state. To explain the magnetization response and particle rotation in the steady state, a transient state is introduced. In the steady state, there is a phase lag between the magnetization rotation and the particle rotation owing to the field applied in the same phase. An additional magnetization rotation caused by another applied field occurs in the same phase as the particle rotation. Thus, the magnetization and particle rotations occurring in the same phase are due to the fields applied in different phases. After the phase delay associated with the Néel relaxation from the first phase when the field is applied, only magnetization rotation occurs, owing to the increase of the Zeeman energy. Particle rotation also occurs after the phase delay associated with the Brownian relaxation from the magnetization rotation. Thus, in the steady state, particle rotation occurs with magnetization rotation in the same phase because magnetization rotation continues as long as the field is applied. To exclude the magnetization rotation that occurs in the same phase as the particle rotation and show the magnetization and particle responses to the field applied at one phase, in the transient response, a field is applied at only phase (i) and is absent at phases (ii) and (iii) (Figure 6a). Phase (i) in the transient state is equivalent to the first phase when the field is applied in the steady state.

There are two types of magnetization rotations, which are distinguished by their occurrence mechanisms. Type I and II magnetization rotations are caused by increasing Zeeman energy and decreasing anisotropic energy due to particle rotation, respectively. The magnetization rotates to the direction in which the potential energy *E* = *E*_a_ − *E*_h_ is lowest (Equations (7) and (8)). Particle rotation is caused by the magnetic torque derived from type I magnetization rotation. Dieckoff et al. reported that both the relaxation times decrease with increasing field intensity, which indicates that the magnetization and particle rotations are due to the applied field, as shown in Figure 6 [8].

In the transient response (Figure 6a), the Zeeman energy is increased by applying a field (phase (i)). A phase lag associated with the Néel relaxation occurs after phase (i), type I magnetization rotation due to the increase of the Zeeman energy in phase (i) occurs, and a magnetic torque is generated by this magnetization rotation (phase (ii)). Moreover, the phase lag associated with Brownian relaxation occurs after phase (ii), the particle rotates owing to the magnetic torque in phase (ii), and type II magnetization rotation occurs because of the decrease in the anisotropic energy expressed by Equation (8) (phase (iii)). This is because the angle between the easy axis and the magnetization decreases owing to the particle rotation. In the steady-state response (Figure 6b), type II magnetization rotation (Figure 6(b-2)) due to particle rotation (Figure 6(b-1)) coexists with type I magnetization rotation (Figure 6(b-3)) because the field is continuously applied. As with the transient response, type I magnetization rotation (Figure 6(b-3)) is derived from the applied field. There is the phase lag associated with Néel relaxation between type I magnetization rotation and the applied field. Particle rotation (Figure 6(b-1)) is derived from type I magnetization rotation. There is the phase lag associated with Brownian relaxation between type I magnetization and the particle rotation. Type II magnetization rotation is induced by the particle rotation. Thus, type I and type II magnetization rotations coexist in the same phase but are caused by the field applied at different phases. 

## 4. Conclusions

We showed, using measured AC hysteresis loops, that Brownian relaxation, which is slower than Néel relaxation, can occur after Néel relaxation when both relaxations coexist. This causes a discrepancy between theoretical predictions and measurements of a real fluid, with regard to magnetic-relaxation properties such as the frequency and viscosity dependences of the magnetization and the phase delay. In the real magnetic fluid, Brownian and Néel relaxations are influenced by each other. The magnetization rotation causes the particle rotation owing to the magnetic torque, and the particle rotation induces another type of magnetization rotation because of the decrease in the anisotropic energy.

## Figures and Tables

**Table 1:** Theoretical and measured peak frequencies and relaxation times. The theoretical values for Brownian and Néel relaxations were calculated using Equations (1), (2) and (4). The anisotropic constant is 11 kJ/m^3^. The measured values for the Brownian relaxation were estimated according to Figure 2.

Brownian Relaxation					Néel Relaxation	
Viscosity (mPa·s)	Frequency (kHz)		Relaxation Time (ms)		Frequency (MHz)	Relaxation Time (ns)
	Theoretical	Measured	Theoretical	Measured	Theoretical	Theoretical
2.4	1.2	4.6	0.13	0.035	25	6.5
4.7	0.63	1.6	0.25	0.10		
12	0.25	0.60	0.64	0.27		
93	0.032	0.084	5.0	1.8		
